# Small Interfering RNA Therapy for the Management and Prevention of Hypertension

**DOI:** 10.1007/s11906-025-01325-8

**Published:** 2025-01-14

**Authors:** Liwei Ren, A. H. Jan Danser

**Affiliations:** 1 Department of Pharmacy, The Second Clinical Medical College, The First Affiliated Hospital, Shenzhen People’s Hospital, Jinan University, Southern University of Science and Technology), Shenzhen, China; 2 Division of Vascular Medicine and Pharmacology, Department of Internal Medicine, room EE1418b, Erasmus MC, Wytemaweg 80, 3015 CN Rotterdam, The Netherlands

**Keywords:** Small Interfering RNA (siRNA), Zilebesiran, Hypertension, Angiotensinogen

## Abstract

**Purpose of Review:**

To review currently existing knowledge on a new type of antihypertensive treatment, small interfering RNA (siRNA) targeting hepatic angiotensinogen.

**Recent Findings:**

Targeting angiotensinogen synthesis in the liver with siRNA allows reaching a suppression of renin-angiotensin system (RAS) activity for up to 6 months after 1 injection. This might revolutionize antihypertensive treatment, as it could overcome non-adherence, the major reason for inadequate blood pressure control. Animal data support that its effects on blood pressure and end-organ damage are fully comparable to those of classical RAS blockers, and phase I and II clinical trials confirm its antihypertensive effectiveness and long-term action. Although its side effect profile is placebo-like, its long-term effects also pose a threat in patients who require immediate restoration of RAS activity, like in shock. Here tools are being developed, called REVERSIR, that allow immediate annihilation of the siRNA effect in the liver.

**Summary:**

One subcutaneous injection of angiotensinogen siRNA lowers blood pressure for 6 months without severe side effects. The decrease in angiotensinogen and blood pressure can be reversed with a drug called REVERSIR if needed.

## Introduction

Hypertension is the leading cause of cardiovascular disease and premature death worldwide. Its prevalence in adults is around 30% (1.4 billion people) [1]. Inadequate blood pressure control is a major cause of stroke, heart failure, and kidney disease, and is driven by many factors, including nonadherence. Non-adherence in hypertension ranges from 47% based on pill-counting to 56% based on self-reporting [2]. Obvious reasons for non-adherence are side effects and the tablet burden, i.e. taking drugs as a lifelong daily treatment without immediate effect. Improving adherence is vital to destroy this vicious circle. Although electronic health tools may improve adherence [3], a long-acting drug that does not require daily dosing would be a potential solution. Here it is important to note that, normally, nocturnal blood pressure should come down by about 10–20% compared with daytime levels [4]. The absence of such dipping, occurring particularly with short-acting drugs, associates with adverse cardiovascular outcomes [4]. Also from this perspective, a longer acting drug might be advantageous. Currently, small interfering RNA (siRNA) drugs might offer such long-term effects. Up to now, 6 siRNA agents have been approved by the FDA: patisiran, givosiran, lumasiran, vutrisiran, inclisiran and nedosiran [5–10] (Table 1). Initially, they were developed for rare diseases, but now their application is shifting to more common diseases, exemplified by the approval of inclisiran for hypercholesterolemia in 2020 [11]. Zilebesiran is an siRNA targeting hepatic angiotensinogen, meant for the treatment of hypertension. Recent data from a phase II clinical trial indicate that it is well tolerated, has a long half-life (one injection is required per 6 months) and reduces blood pressure substantially [12]. This review focuses on this novel drug, discussing why one might target angiotensinogen, how the long-lasting effect can be achieved, and summarizes the first preclinical and clinical data, while ending with a description of its risks and side effects, and what to do about them.

**Table 1:** Currently available small interfering RNA therapies

Drug	Condition	Administration	Time of approval	Company	Target	Modification
patisiran	polyneuropathy of hereditary transthyretin-mediated amyloidosis	intravenous infusion	08/2018	Alnylam Pharmaceuticals	hepatic hATTR	lipid nanoparticles
givosiran	acute hepatic porphyria	subcutaneous	11/2019	Alnylam Pharmaceuticals	hepatic ALAS1	GalNAc
lumasiran	primary hyperoxaluria type 1	subcutaneous	11/2020	Alnylam Pharmaceuticals	hepatic HAO1	GalNAc
inclisiran	primary hyperlipidemia, including heterozygous familial hypercholesterolemia	subcutaneous	12/2021	Novartis	hepatic PCSK9	GalNAc
vutrisiran	polyneuropathy of hereditary transthyretin-mediated amyloidosis	subcutaneous	06/2022	Alnylam Pharmaceuticals	hepatic hATTR	GalNAc
nedosiran	primary hyperoxaluria type 1	subcutaneous	09/2023	Novo Nordisk	hepatic LDH	GalNAc

### Why Target Angiotensinogen?

**Fig. 1:**
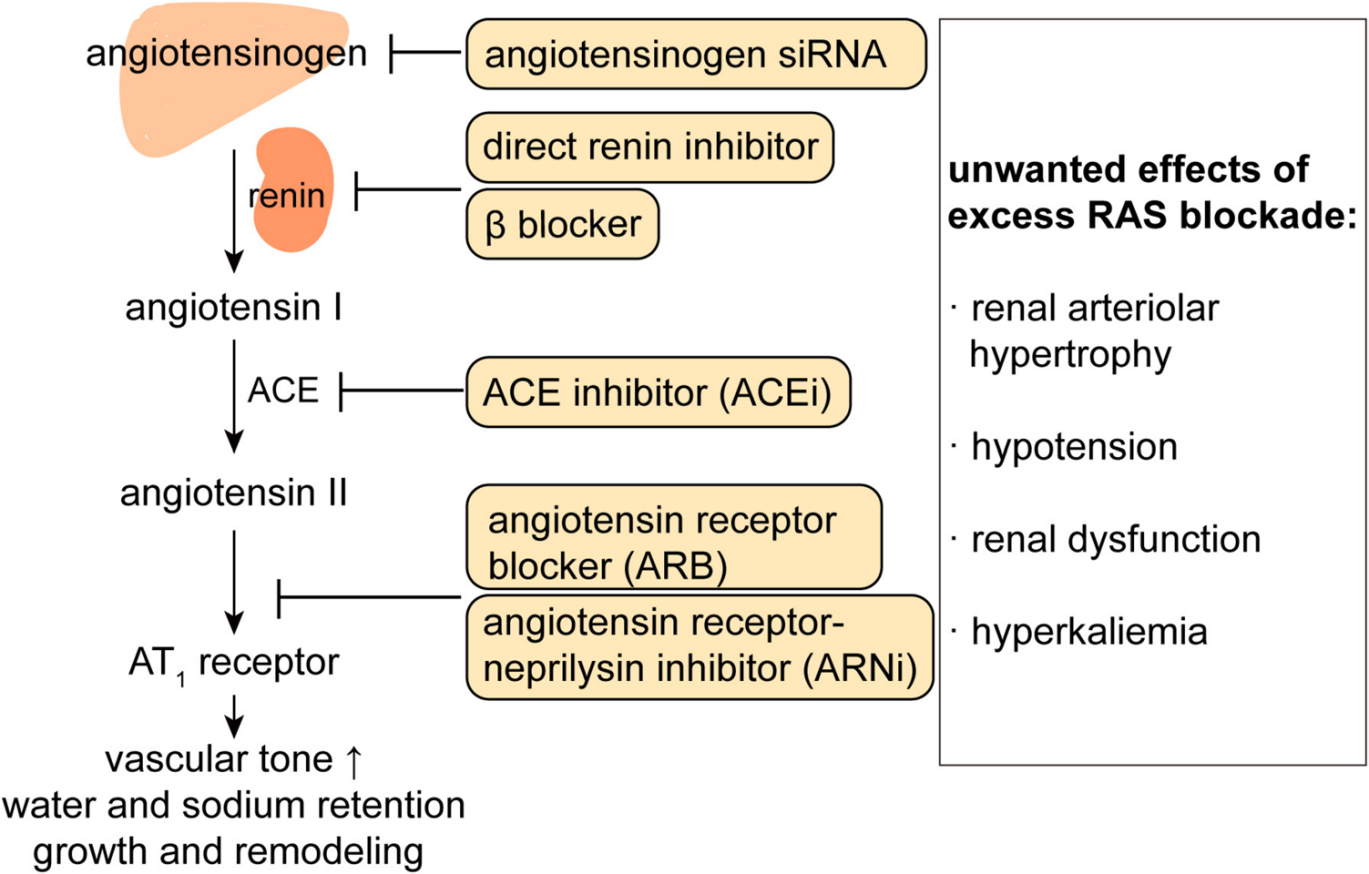
The renin-angiotensin system, the various tools with which it can be blocked, and the potential unwanted consequences of such blockade

The renin-angiotensin system (RAS) is a critical hormonal system that regulates vascular tone, fluid, and electrolyte homeostasis, as well as cardiac and vascular remodeling. For this reason, it has been the target of multiple antihypertensive drugs (Fig. 1), i.e., β blockers (which lower renin release), direct renin inhibitors, angiotensin-converting enzyme (ACE) inhibitors and angiotensin (Ang) II type 1 (AT_1_) receptor blockers [13, 14]. Angiotensinogen, exclusively cleaved by renin to Ang I, is the only substrate from which Ang II is derived. A recent suggestion that the octapeptide Ang II might also be generated by non-renin enzymes from a smaller, 12 amino-acid precursor, Ang-(1–12), turned out not to be true [15]. Ang I is converted by ACE to Ang II, which then exerts its effects via binding to AT_1_ receptors. This includes the release of aldosterone from the adrenal. Like many hormonal systems, the RAS contains a negative feedback loop, allowing AT_1_ receptor stimulation to suppress renin release. This prevents upregulation of the system, but also implies that blocking the system at the level of renin, ACE or the AT_1_ receptor will cause an increase in renin release, allowing the body to restore at least a certain degree of RAS activity. It is important to note that the capacity of the body to upregulate renin is almost infinite, as rises of many 100-fold have been described [16], resulting in renin levels that normally only occur in patients with renin-producing tumors [17]. This is due to the fact that long-term RAS blockade upregulates the number of renin-producing cells [18]. As an example, if achieving 99% renin or ACE inhibition, a 100-fold rise in renin would fully normalize Ang II formation. This phenomenon is often referred to as ‘RAS escape’. As a consequence, the degree of inhibition may not as extensive as aimed for [19]. The reason that renin rises allow such a rapid and easy return of Ang II formation is that our angiotensinogen levels (around 1200 pmol/mL) are > 1 million-fold higher than those of Ang II (fmol/mL range) [20]. Thus, any renin rise will immediately translate in a parallel rise in angiotensin formation. From this point of view, targeting angiotensinogen might be a logical choice to avoid the RAS escape. Angiotensinogen levels are generally stable, but increase 2–3-fold during pregnancy [21], due to the fact that estradiol upregulates angiotensinogen expression, and decrease under conditions of severe renin upregulation, like in patients with heart failure treated with diuretics and RAS blockers [22, 23].

Although the majority of angiotensinogen synthesis classically occurs in the liver, for decades it has been thought that angiotensinogen is also synthesized at extrahepatic sites, like the kidney, heart, adipose tissue and brain [24–27]. This is important, since Ang II generation at the tissue level is thought to be of much greater importance than Ang II generation in blood [28, 29]. However, selective targeting of hepatic angiotensinogen has challenged this view. It revealed that deleting angiotensinogen in the liver also eliminated the angiotensinogen protein (but not its mRNA) in kidney, heart and adipose tissue [20, 30–32]. This leaves only the brain as a potential extrahepatic angiotensinogen synthesis site. Nevertheless, even at this site Ang II disappeared after deleting liver angiotensinogen [32], implying that brain angiotensinogen synthesis may not be functional, for instance because it remains intracellular, or because renin is simply lacking [15, 33]. Possibly, Ang II in the brain is blood-derived, and not locally synthesized [32–34]. Taken together, it seems that selectively targeting hepatic angiotensinogen is sufficient to suppress angiotensin generation in the entire body, i.e., tissue angiotensin generation relies on hepatic angiotensinogen. Thus, the effects of hepatic angiotensinogen suppression should minimally mimic those of classical RAS blockers. Moreover, they might be hampered to a lesser degree by the RAS escape.

### Small Interfering RNA as a New Treatment Tool

DNA and RNA are fundamental for protein synthesis and activity [35], and interfering at the level of RNA will prevent protein synthesis. This offers the possibility to also interfere at the level of proteins that cannot be blocked by using either an enzyme inhibitor or a receptor antagonist (as currently most drugs do). Several therapeutic approaches targeting RNA have been developed, including antisense oligonucleotides (ASO) and siRNA [19]. ASO-based therapies utilize Watson-Crick’s base-pairing rules and single-strand DNA containing 15–30 nucleotides, which are designed in anti-sense orientation to the pre-mRNA and mRNA of interest [36]. When the ASO binds to the target pre-mRNA, RNase H1 cleaves the RNA in a DNA-RNA duplex in both the cytoplasm and nucleus [37], destroying the mRNA and inhibiting the translation of the targeted protein.

**Fig. 2:**
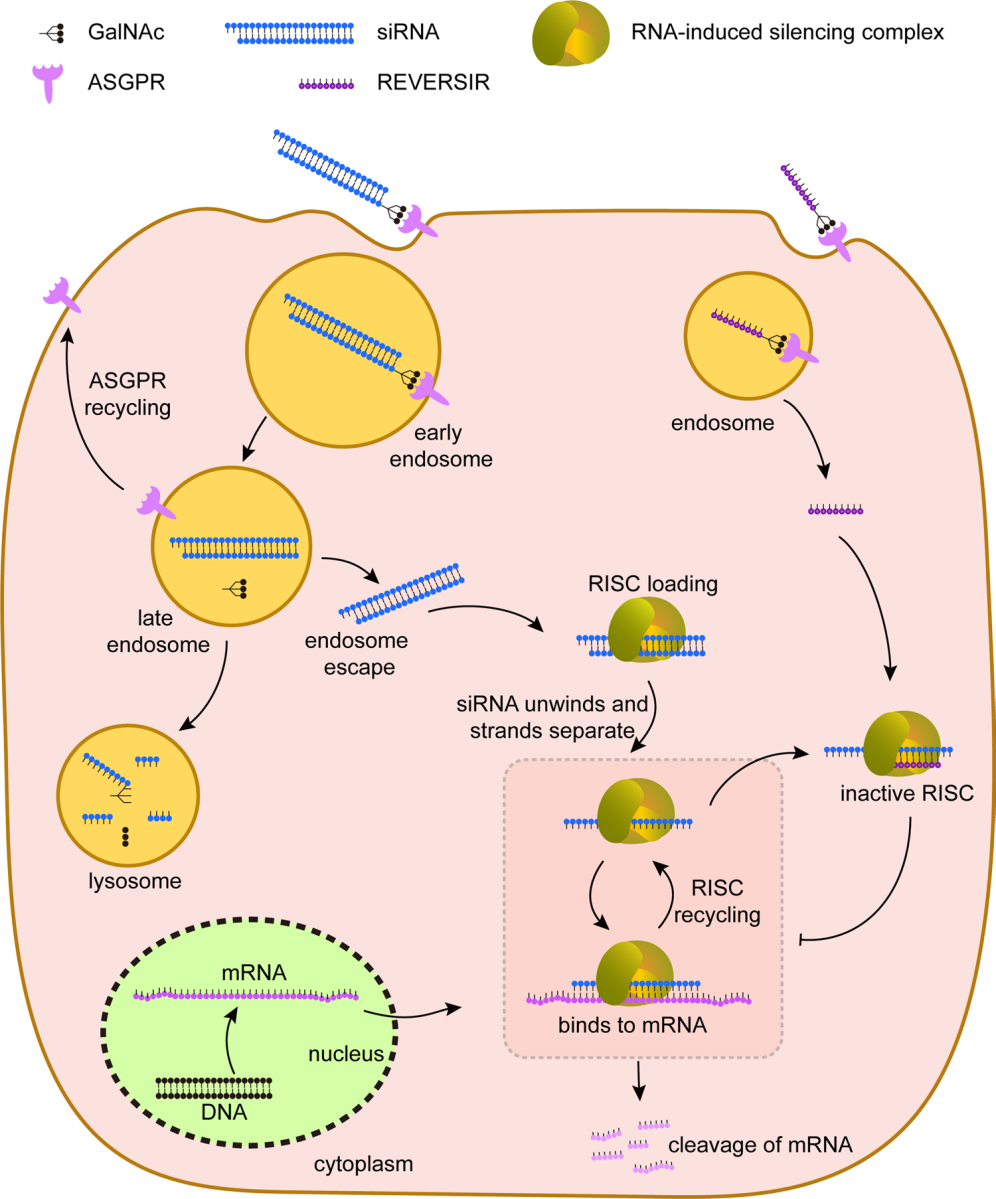
The mechanism of action of GalNAc-conjugated small interfering RNA (siRNA) to silence hepatocyte genes and the mechanism of REVERSIR to reverse the siRNA effect, thus preventing the cleavage of targeted mRNA. ASGPR, asialoglycoprotein receptor; GalNAc, trivalent N-acetylgalactosamine

In contrast to the ASO approach, siRNA utilizes double-strand RNA, named passenger strand and guide strand, regulating gene expression in an evolutionary conserved mechanism [38]. The siRNA duplexes are loaded into a multiprotein complex called RNA-Induced Silencing Complex (RISC). Next, the passenger strand is discarded, while the guide strand remains, which then base-pairs with its complementary sequences in the target mRNA [39]. Argonaute, the core catalytic protein of RISC [40], cleaves the mRNA precisely at the paired site, resulting in the degradation of targeted mRNA (Fig. 2). Chemical modification and different delivery systems have been addressing challenges of translation of siRNA to the clinic, including rapid renal clearance, degradation by ubiquitous RNases, difficulties in delivery across the cell membrane, risks of off-target gene silencing, and immune-mediated toxicity [41, 42].

All 6 currently approved siRNA drugs target the liver, suppressing the synthesis of proteins that are largely, if not exclusively, liver-derived. Only the first (patisiran, used for the treatment of hereditary transthyretin-mediated amyloidosis) is formulated as lipid nanoparticles, while the other five siRNAs are modified by trivalent N-acetylgalactosamine (GalNAc), which results in liver-specific delivery. The reason for this is that GalNAc is a peptide ligand that binds to hepatocytes via the so-called asialoglycoprotein receptor, which is abundantly expressed in these cells (> 10^6^ copies/hepatocyte) [43, 44]. This mechanism can carry up to 10^6^ siRNAs into the cell every 10–15 min. The GalNAc conjugation promotes the efficacy of hepatocyte delivery 10 times [45], and 4 weeks after the subcutaneous administration of GalNAc-siRNA, its hepatic accumulation is up to 50 times above that in the kidney [46]. The remarkable durability of GalNAc-siRNA (more than 6 months in humans) relates to its accumulation and stability in acidic intracellular compartments, which allows the release of functional siRNA and loading into Argonaute protein even many weeks after a single application [12, 47]. As such, siRNA in this acidic compartment might be considered as a depot, resulting in continuous RISC loading and thus long-lasting gene-silencing [42].

ASOs display a much shorter half-life than siRNAs, and thus only the latter appears particularly suitable to improve patient adherence [48]. In clinical studies, both ASO and siRNA have utilized GalNAc conjugation to target hepatic angiotensinogen mRNA. Compared with GalNAc-ASO, a single dose of GalNAc-siRNA was capable of deleting plasma angiotensinogen by > 90% for up to 6 months, while GalNAc-ASO maximally reduced plasma angiotensinogen by 60% for one week [12, 49]. As a consequence, the use of angiotensinogen ASO for the treatment of hypertension has currently been abandoned.

### Angiotensinogen siRNA Effects in Animals and Humans

Studies in animals have provided detailed mechanistic insights into the effectiveness of angiotensinogen GalNAc-siRNA [32, 46, 50–52] (Table 2). At a 10 mg/kg dose, it eliminated circulating angiotensinogen in spontaneously hypertensive rats (SHR) by 98%, yet without suppressing circulating Ang II. It did suppress Ang II in tissues, and this therefore most likely explained why it reduced blood pressure to the same degree as classical RAS blockers like valsartan. Here it is important to mention that rats, like humans, display circulating angiotensinogen levels in the K_m_ range (i.e., around 1 µmol/L). This contrasts with mice, where circulating angiotensinogen levels are only about 1% of those in humans and rats. Nevertheless, mice have the same angiotensin levels as humans and rats [20]. This is possible due their exceptionally high renin levels, and illustrates that in mice small changes in angiotensinogen may have substantial consequences, while in humans and rats regulation occurs rather at the level of renin. However, when suppressing angiotensinogen by 98%, the rat circulating RAS becomes more like that in mice, particularly because renin will rise. This explains why, at least in blood, Ang II levels could stay normal, even when only 2% of angiotensinogen remained. Yet, the angiotensinogen delivery at tissue sites now became insufficient, and thus tissue Ang II levels did go down. In other words, the RAS escape only occurred in the circulation, and not at the tissue level. Interestingly, combining angiotensinogen siRNA and valsartan reduced angiotensinogen even further (by 99.8%), and now both circulating and tissue Ang II became virtually undetectable [46]. This reflects the even bigger renin rise during their combined application, apparently resulting in rapid cleavage and disappearance of the small remaining quantities of angiotensinogen. As a consequence, mean arterial blood pressure dropped to a much greater degree (≈70 mm Hg) than when each drug was given alone (≈20–25 mm Hg), implying synergy. Such impressive synergy is only expected when truly annihilating angiotensinogen, and will not necessarily occur when combining RAS blockers at doses that only partially suppress angiotensinogen, since under such circumstances the elevated renin levels are still capable of generating sufficient Ang II.

**Table 2:** Potential consequences of angiotensinogen small interfering RNA

effects	Rat				Human		
	SHR	DOCA-salt	5/6th nephrectomy	diabetic TGR(mRen2)27	mild-to-moderate hypertension	obese hypertension	hypertension with cardiovascular disease or advanced chronic kidney disease
eliminating angiotensinogen	√	√	√	√	√	ongoing	ongoing
blood pressure lowering	√	×	√	√	√	ongoing	ongoing
cardio-protection	√	√	√	√	---	---	ongoing
renal protection	---	---	√	√	---	---	ongoing

*SHR*, spontaneously hypertensive rat. *TGR*, transgenic rat

As expected, angiotensinogen siRNA did not reduce blood pressure in models where the RAS is suppressed (like to deoxycorticosterone-salt rat), while it induced reno- and cardio-protection in rats displaying chronic kidney disease (5/6th nephrectomy model), diabetic kidney disease and/or cardiac hypertrophy [32, 50, 51]. These beneficial end-organ effects occurred, at least partially, in a blood pressure-independent manner, suggesting interference with the generation of Ang II at the tissue level. Indeed, in all models, angiotensinogen siRNA lowered the renal and cardiac Ang II levels, illustrating that angiotensin generation at these sites relies entirely on hepatic angiotensinogen. It did not affect glomerular filtration. The effects on blood pressure and tissue damage were comparable to those of classical RAS blockers like the ACE inhibitor captopril and the AT_1_ receptor antagonists valsartan or losartan, and when combining 2 blockers (e.g., valsartan + angiotensinogen siRNA), the effects were even stronger. Clearly, these data demonstrate that angiotensinogen siRNA is at least as effective as a classical RAS blocker, and exerts the same beneficial effects in kidney and heart. The difference of course is that this does not require daily dosing.

In a human phase I trial, zilebesiran dose-dependently suppressed plasma angiotensinogen in hypertensive patients, and the effects of one subcutaneous injection remained apparent for up to 6 months [12]. Blood pressure decreased in parallel, and the largest effects were obtained at the highest zilebesiran dose (800 mg). Blood pressure lowering was larger with a low-salt diet than a high-salt diet, in full agreement with the well-known fact that a high-salt diet suppresses the RAS. Providing irbesartan on top of zilebesiran induced further lowering of blood pressure. The results of three phase II trials are expected in the coming years. KARDIA-1 investigates the effects of various zilebesiran doses (150–600 mg) in patients with mild-to-moderate hypertension not receiving other antihypertensive drugs [53], while KARDIA-2 will study the effect of 600 mg zilebesiran in such patients when combined with a diuretic (andapamide), a calcium antagonist (amlodipine) or an AT_1_ receptor antagonist (olmesartan) [54]. The KARDIA-3 protocol, presented in May 2024 at the European Society of Hypertension meeting in Milan, will evaluate the efficacy and safety of zilebesiran (150–600 mg) as add-on therapy in patients who have established cardiovascular disease or high cardiovascular risk with or without advanced chronic kidney disease, and hypertension that is uncontrolled despite stable treatment with 2–4 antihypertensive drugs [55]. The 3- and 6-month results of KARDIA-1 have been published [53]. After 3 months, the effects of 150, 300 and 600 mg were comparable (a drop of 7.3, 10, and 8.9 mm Hg in ambulatory systolic blood pressure, respectively), while at 6 months the effects of 150 mg angiotensinogen siRNA (6.5 mm Hg) tended to be smaller than that of the other doses (9.5–9.6 mm Hg). The first results of KARDIA-2 were presented at the 2024 American College of Cardiology meeting [54], and demonstrated that the add-on effects of zilebesiran were largest on top of indapamide, and smallest with olmesartan. This confirms current clinical practice with RAS blockers, and illustrates that the diuretic-induced increase in renin release will enhance the response to a RAS blocker. The modest additional effects on top of olmesartan contrasts with the synergistic drop in blood pressure when applying angiotensinogen siRNA together with valsartan in SHR. To fully understand these data, one needs to know the degree of angiotensinogen suppression and renin upregulation. A likely explanation is that, in patients, angiotensinogen was not fully suppressed, allowing at least a certain degree of angiotensin generation, and not its entire disappearance like in the rats [55].

### Safety Aspects of Angiotensinogen siRNA

Given that zilebesiran is an entirely new type of RAS blocker, utmost care should be taken to map its side effects in detail before its introduction on the market. At present, its best comparator might be inclisiran, which relies on the same GalNAc principle, but once present in the hepatocyte targets proprotein convertase subtilisin/kexin type 9 [56] instead of angiotensinogen. Like zilebesiran, a single subcutaneous injection of inclisiran lasts 6 months. It lowered LDL levels by about 50%. Its only adverse event until now seems to be the injection-site reaction, occurring in 3–5% of the trial participants [10, 12, 56, 57]. No liver toxicity or inflammatory and immunologic side effects were reported, confirming that liver accumulation of this type of siRNA is not by definition hepatotoxic. KARDIA-1 also reported no serious hepatic events, and at most some mild, non-serious and transient hypotension (4% of patients) and hyperkaliemia (6%), as well as the injection-site reaction (6%) [53]. Of course, it is well-known that too much RAS blockade might result in hypotension, renal dysfunction and hyperkaliemia. This will particularly occur when applying more than one RAS blocker [58]. It reflects the fact that normal kidney function (in particular glomerular filtration) does require certain degree of RAS activity. The ongoing KARDIA studies, when higher patient numbers have been reached, will shed further light on this issue, and it is clear that full annihilation of angiotensinogen is not desirable. Moreover, a high degree RAS inhibition associates with concentric thickening of the renal arteries and arterioles due to expansion of immature matrix-producing renin cells and inward accumulation of abnormal smooth muscle cells [18]. Recent animal data show that this is also the case during angiotensinogen siRNA treatment. Reassuringly, it was fully comparable to that seen during ACE inhibition and AT_1_ receptor antagonism [59].

A final concern about angiotensinogen siRNA might be its long duration of action. Of course, this is very helpful with regard to adherence, but could become a problem when the RAS is acutely needed to maintain blood pressure and to prevent acute kidney injury, like under conditions of sepsis and hemorrhage [60]. Another reason requiring a rapid return to normal RAS activity is pregnancy, as the RAS is a major player in the hemodynamic alterations occurring this condition [61, 62]. Preclinical studies in SHR on a low-salt diet, responding strongly to angiotensinogen siRNA, revealed that acutely, bolus injections of both Ang II and norepinephrine could increase blood pressure, while chronically a high salt diet and fludrocortisone allowed blood pressure to return to normal, although this took several days [30].

A more elegant approach, termed REVERSIR, is to reverse the siRNA-induced gene silencing [63]. This technology involves synthetic single-stranded oligonucleotides that are complementary to the siRNA guide strand loaded in RISC and that target hepatocytes using the same GalNAc delivery approach. Upon binding with high affinity to the RNA-induced silencing complex-loaded siRNA guide strand, REVERSIR blocks recognition and silencing of the corresponding mRNA and, therefore, allows resumption of protein production (Fig. 2). When applying REVERSIR to angiotensinogen siRNA-treated SHR, Ye et al. observed that it allowed the full return of angiotensinogen in a dose-dependent manner within a few days [52]. This was accompanied by a return of blood pressure to its baseline levels. Interestingly, the latter also occurred at a REVERSIR dose that only increased angiotensinogen by 50%. The fact that the renin levels at this dose were still 2–3-fold elevated (thus allowing the same degree of Ang II generation as occurred at baseline, despite angiotensinogen being 50% lower) explains why blood pressure might have returned to normal. This illustrates that even a partial normalization of angiotensinogen might be helpful in patients to rapidly increase blood pressure.

## Conclusion and Perspectives

Angiotensinogen siRNA or zilebesiran is a promising drug for the treatment of hypertension. It additionally exerts reno- and cardio-protection. Importantly, its effects are fully comparable to those of classical RAS blockers like the ACE inhibitors and AT_1_ receptor antagonists. Yet, it only requires one injection every 6 months, and thus might greatly improve adherence, which is the major reason for inadequate blood pressure control. Its side effect profile is placebo-like, and at most its long-term effects pose a risk in patients requiring acute restoration of RAS activity, like during shock. Tools already exist to acutely reverse its effects if needed. Extensive preclinical and clinical programs are on the way to investigate its efficacy and safety profile.

## Data Availability

No datasets were generated or analysed during the current study.
